# Differential Enzymatic Hydrolysis: A Study on Its Impact on Soy Protein Structure, Function, and Soy Milk Powder Properties

**DOI:** 10.3390/foods14050906

**Published:** 2025-03-06

**Authors:** Qian Li, Baoyue Chang, Guo Huang, Di Wang, Yue Gao, Zhijun Fan, Hongbo Sun, Xiaonan Sui

**Affiliations:** 1 Heilongjiang Green Food Science Research Institute, Harbin 150028, China; 2 College of Food Science, Northeast Agricultural University, Harbin 150030, China

**Keywords:** enzymatic hydrolysis, soy protein isolate (SPI), soy milk powder, structure, physicochemical properties

## Abstract

Protein constitutes the primary nutrient in soy, and its modifications are intricately linked to the properties of the soy milk powder. This study employed six main commercial enzymes (bromelain, neutrase, papain, trypsin, flavourzyme, and alcalase) to investigate the impact of enzymatic hydrolysis on the structural and functional properties of soy protein isolate (SPI), as well as its influence on the physicochemical properties of soy milk powder. The findings indicated that each of enzymes exhibits distinct specificity, with the degree of hydrolysis following the order: alcalase > flavourzyme > papain > bromelain > neutrase > trypsin. Enzymatic hydrolysis facilitates the unfolding of SPI, leading to the exposure of chromogenic fluorophores and hydrophobic amino acid residues, which in turn promotes an increase in free sulfhydryl content. Concurrently, this process induces the transformation of *α*-helix and *β*-sheet into *β*-turn and random coil. The enzyme modification enhances the solubility, emulsification, and foaming activities of SPI and significantly augment its antioxidant properties (*p* < 0.05). However, this enzymatic treatment adversely affects the stability of its emulsification and foaming properties. Subsequent to enzymatic hydrolysis, soy milk powder demonstrated a reduction in particle size and an improvement in solubility, which significantly enhanced its flavor profile. In summary, alcalase offers substantial advantages in augmenting the functional properties of SPI and increasing the solubility of soy milk powder. However, this process adversely affects the flavor profile of soy milk powder, a consequence attributed to the broad hydrolysis specificity of alcalase.

## 1. Introduction

Soy protein, a high-quality protein, is the main bioactive substances in soy and has been widely used in food industry to formulate various healthy diets for nutritional demand in recent years. The majority of soy protein is ingested as soy powder, soy protein concentrate, and soy protein isolate (SPI), which are incorporated into food products in a refined form to enhance product characteristics and nutritional composition during the production of processed foods. These enhancements include increased gelation, emulsification, and other advantageous functions [1]. Soy milk is recognized for its advantageous properties associated with the bioactive compounds present in soybeans, particularly soy saponins and soy protein. Furthermore, soy milk is characterized by a high concentration of lecithin and vitamins, as well as the presence of isoflavones, which are potent antioxidants. Consequently, the health benefits of soy milk are augmented by the physiological effects of its constituent functional compounds. Properly processed soy milk and its derivatives provide a range of nutraceutical and health benefits [2]. Soy milk powder is an edible powder obtained by spray drying soy milk, which is rich in iron, unsaturated fatty acids and niacin. Soy milk powder has been extensively investigated as a promising economical substitute for dairy products because of decreasing for occurrence of lactose intolerance of soy milk powder free from lactose. However, during the spray drying process, the high temperature-induced protein denaturation may act as a barrier for the solubility of soy milk powder and consequently restrict its powdered beverage applications in the food industry. Furthermore, the poor solubility of soy milk powder could also lead to the difficulties in processing, resulting in a little acceptable taste and a decrease in nutritional value [3]. More, certain technical challenges persist in the incorporation of nutrients and bioactive compounds into soy milk. Specifically, issues related to low solubility and bioavailability must be addressed to ensure optimal delivery [4].

As the main component of soy milk powder, the properties of soy protein may be closely related to the functional properties of soy milk powder. Enzymatic hydrolysis has been shown to improve the functional properties of protein, including solubility, emulsification and foaming properties, which is considered to be an effective method to improve the sensory characteristics and nutritional components of food [5]. Moreover, different enzymes have different effects on the functional properties of proteins, which may be determined by the specificity of different enzymes. In this technique, enzymatic hydrolysis by a site-specific enzyme cleaves proteins into a different mixture of peptides and free amino acids, thereby increasing hydrophilicity and improving functional properties [6]. For instance, compared with papain and trypsin, Tang et al. pointed out that treatment with subtilisin resulted in the highest solubility and stability coefficient, possibly due to the enzyme’s active site [7]. Compared with microbial enzymes and alcalase, flavorzyme have a higher degree of hydrolysis. For this reason, more low molecular weight peptides can be generated from SPI through gradual hydrolysis of flavourzyme, thereby significantly improving protein solubility and other functional properties [8]. Moreover, it can be deduced that enzymatic hydrolysis may enhance the functional properties of soy milk powder [9]. The majority of research has concentrated on examining the impact of select enzymes, such as bromelain, on the functional properties of proteins [10]. However, to the best of our knowledge, there are few studies on the effects of different enzymes on the functional properties of soy milk powder under enzymatic hydrolysis.

**Figure 1:**
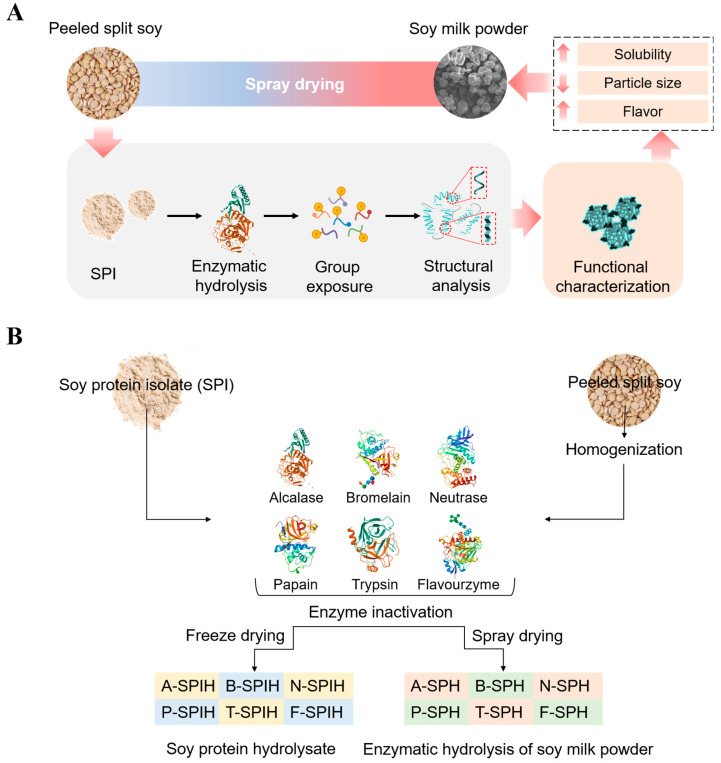
Study flow diagrams for the study topics (**A**), and flowchart for preparation in the study of soy protein hydrolysate and enzymatic hydrolysis of soy milk powder (**B**).

Therefore, we propose that the properties of soy milk powder could be enhanced through the enzymatic treatment. In this study, soy protein hydrolysate was generated at varying degrees of hydrolysis using six widely available commercial enzymes: bromelain, neutrase, papain, trypsin, flavourzyme, and alcalase. The alterations in the secondary and tertiary structures of SPI were systematically examined. Subsequently, these enzymatic treatment were applied to the production of soy milk powder to improve its properties. The mechanism by which soy milk powder enhances properties was subsequently examined in terms of the degree of hydrolysis, as well as the structural and functional changes in SPI that were preserved (Figure 1A). This investigation aims to propose a novel strategy for improved bioactive delivery by exploring enzymatic hydrolysis of soy protein.

## 2. Materials and Methods

### 2.1. Materials

SPI was provided by Shandong Fuhe Biotechnology Co., Ltd. (Jinan, China). Bromelain (enzyme activity 6.0 × 10^5^ U/g, optimum temperature 30–45 °C, optimum pH 6.0–6.8), neutrase (enzyme activity 5.0 × 10^4^ U/g, optimum temperature 50–55 °C, optimum pH 6.0–7.0), papain (enzyme activity 8.0 × 10^5^ U/g, optimum temperature 50–55 °C, optimum pH 6.0–7.0), trypsin (enzyme activity 2.5 × 10^5^ U/g, optimum temperature 37 °C, optimum pH 7.5–8.0), flavourzyme (enzyme activity 3.0 × 10^4^ U/g, optimum temperature 50 °C, optimum pH 7.0–7.5), alcalase (enzyme activity 2.0 × 10^5^ U/g, optimum temperature 50 °C, optimum pH value 9.0–11.0), which were acquired from Beijing Solebaugh Technology Co., Ltd. (Bejing, China). Peeled split soy was provided by Heilongjiang Beidahuang Green Health Food Co., Ltd. (Jiamusi, China). All other chemicals used were of analytical grade.

### 2.2. Preparation of Soy Protein Hydrolysate and Determination of Hydrolysis Degree

Soy protein hydrolysate was prepared using a previously described method with some modifications [11]. Briefly, the assay was performed at optimum temperature and pH according to Section 2.1, in which, the selection of relevant temperature and pH value is based on the median selection of their applicable range. Before testing, the enzymatic activities of various enzymes were determined. SPI powder was dispersed in deionized water (1.0%, *w*/*v*) in equal parts and the mixture was let to magnetically stir for 2 h with a stirring speed of 150 rpm to allow sufficient dissolution. After that, the 3000 U/g of protein enzymes was added thereto for hydrolysis for 15 min, during which the pH was kept constant with using 0.1 M HCl or NaOH solution. Subsequently, the resulting soy protein hydrolysate was heated in an 85 °C water bath for 10 min to terminate the reaction, and immediately cooled to room temperature in an ice water bath. Therefore, the pH was adjusted to 7.0, and then centrifuged for 20 min (3000× *g*, 4 °C). The supernatant was freeze-dried to final obtain trypsin hydrolysate (T-SPIH), neutrase hydrolysate (N-SPIH), bromelain hydrolysate (B-SPIH), papain hydrolysate (P-SPIH), flavourzyme hydrolysate (F-SPIH) and alcalase hydrolysate (A-SPIH), which were ground and refrigerated for later use (Figure 1B). SPI that was not subjected to enzymatic treatment was considered control sample.

The degree of hydrolysis (DH) was determined by the *o*-phthalaldehyde (OPA) method as described by Nielsen et al. using serine as the standard [12].

### 2.3. Characterization of Structure of Soy Protein Hydrolysate

#### 2.3.1. Sodium Dodecyl Sulfate-Polyacrylamide Gel Electrophoresis (SDS-PAGE) Measurements

For SDS-PAGE measurements, 0.01 M, pH 7.0 phosphate buffered solution (PBS) was used to configure 2 mg/mL of soy protein hydrolysate dispersion and subsequently for 2 h with gentle stirring at room temperature. The reducing gel electrophoresis running conditions were according to the description of Meinlschmidt et al., including a separation gel of 12.0% and a stacking gel of 5.0% [13]. The electrophoresis procedure was conducted within a cold chamber maintained at 4 °C for a duration of 1–2 h. Initially, a voltage of 60 V was applied, which was subsequently increased to 120 V upon entry into the separating gel.

#### 2.3.2. UV Spectrometry Measurements

The 1 mg/mL soy protein hydrolysate dispersion was prepared with PBS (0.01 M, pH 7.0). UV absorption spectra of dispersion was recorded at 200–500 nm by a UV-2600 spectrophotometer (Shimadzu, Kyoto, Japan).

#### 2.3.3. Fluorescence Spectrometry Measurements

The 0.1 mg/mL soy protein hydrolysate dispersion was prepared with PBS (0.01 M, pH 7.0). The emission spectra of the dispersion were performed by using a RF-6000 fluorescence spectrophotometer (Shimadzu, Kyoto, Japan) according to a previous study [4].

#### 2.3.4. Free Sulfhydryl Content Measurements

A dispersion of soy protein hydrolysate at 5 mg/mL was prepared in Tris-Gly buffer at pH 8.0 and stirred for 15 min at room temperature. The dispersion was followed by centrifuged for 15 min (8000× *g*, 4 °C) to collect the supernatant for further protein concentration assay by BCA protein Assay Kit (BCA; Thermo Fisher Scientific, Waltham, MA, USA). To 6 mL of the supernatant, 60 μL of DTNB solution (4 mg/mL) was added and incubated in a dark environment for 15 min. The concentration of free sulfhydryl groups was quantified by UV absorbance at 412 nm using a UV-2600 spectrophotometer (Shimadzu, Kyoto, Japan). The equation is as follows:

(1) $$Free\;sulfhydryl\;content\;\left(\mu M/g\right)=\frac{73.53\times A_{412}\times D}{C}$$

where, *A_412_* is the absorbance at 412 nm; *D* is the dilution factor and *C* is the sample concentration in mg/mL. The constant of 73.53 was derived from 10^6^/(1.36 × 10^4^) where 1.36 × 10^4^ is the molar absorptivity and 10^6^ is used for unit conversion (from molar basis to μM/mL, and from mg to g solids).

#### 2.3.5. Surface Hydrophobicity Measurements

The soy protein hydrolysate powder was dissolved in PBS (0.01 M, pH 7.0) and stirred for 2 h. After centrifugation for 10 min (9500× *g*, 4 °C), a 1 mg/mL hydrolysate dispersion was obtained, which was diluted to different concentration dilutions (0.2, 0.4, 0.6, 0.8 and 1 mg/mL). For details, surface hydrophobicity measurements referred to the method using a RF-6000 fluorescence spectrophotometer (Shimadzu, Kyoto, Japan) described by Tang et al. [5]. Surface hydrophobicity values were determined through linear regression analysis of the slope derived from the relationship between relative fluorescence intensity and protein concentration.

#### 2.3.6. Fourier Transform Infrared (FT-IR) Spectral Measurements

FT-IR spectroscopy was carried out by a Nicolet 6700 FT-IR spectrometer (Thermo Scientific, Waltham, MA, USA) using KBr discs to investigate the secondary structure change of soy protein hydrolysate, which was measured as previously described [4]. Specifically, in total reflectance mode, the adsorption properties were documented between 4000 and 400 cm^−1^ with 32 scans and a resolution of 4 cm^−1^. The PeakFit Version 4.12 software (SPSS Inc., Chicago, IL, USA) was used to calculate the relative secondary structure content of soy protein hydrolysate of amide I band (1700–1600 cm^−1^).

### 2.4. Characterization of Functional Properties of Soy Protein Hydrolysate

#### 2.4.1. Solubility Measurements

The dispersion was formulated by dissolving soy protein hydrolysate powder in PBS (0.01 M, pH 7.0) at a concentration of 10 mg/mL. Following a 30 min stirring period at room temperature, the supernatant was isolated by centrifuging for 20 min (10,000× *g*, 4 °C). The protein content of the supernatant was determined using the BCA protein Assay Kit (BCA; Thermo Fisher Scientific, Waltham, MA, USA). Protein solubility could be expressed as the percentage of soluble protein relative to the total protein amount.

#### 2.4.2. Emulsification and Foaming Properties Measurements

Emulsification and foaming properties were determined according to the method of Sui et al. [14]. Emulsification properties were determined by turbidimetry to determine the emulsifying activity (EAI) and emulsion stability (ESI) of the soy protein hydrolysate, and foaming properties were determined by measuring the foaming capacity (FC) and foaming stability (FS) of the soy protein hydrolysate after high-speed homogenization.

The EAI and ESI were calculated using the following equations:

(2) $$EAI\;\left(m^{2}/g\right)=\frac{2\times 2.303\times A_{0}\times DF}{10000\times \theta \times L\times C}$$

(3) $$ESI\;\left(min\right)=\frac{A_{0}\times 10}{A_{0}-A_{10}}$$

where, *DF* represents the dilution factor (100), *C* denotes the protein concentration (g/mL), *L* signifies the optical path length of the cuvette (1 cm), *θ* indicates the oil phase fraction of the emulsion (0.25), and *A_0_* and *A_10_* correspond to the absorbance of the emulsions at 0 and 10 min, respectively.

The FC and FS were calculated using Equations (4) and (5).

(4) $$\%\;FC=\frac{V_{0}-V}{V}\times 100$$

(5) $$\%\;FS=\frac{{V_{0}-V}_{t}}{V_{0}}\times 100$$

where, *V* refers to the foam volume prior to whipping, *V_0_* denotes the foam volume immediately after whipping at 0 min, and *V_t_* represents the foam volume after whipping at 20 min.

#### 2.4.3. Antioxidant Measurements

According to the method of Wen et al. [15], the antioxidant activity of the soy protein hydrolysate was characterized by measuring the DPPH and ABTS^•+^ free radical scavenging ability.

### 2.5. Preparation of Soy Milk Powder

Peeled split soy was immersed in 80 °C deionized water at a ratio of 1:6 (*w*/*v*), and then 0.7% sodium bicarbonate solid (sodium bicarbonate: dehulled half bean, *w*/*w*) was added and stirred for 1 min. The mixture was ground 3 times using a universal wall breaker and then centrifuged for 3 min (3000× *g*, 25 °C). The supernatant was separated to obtain soy milk. The precipitated dregs were added to 80 °C deionized water at a ratio of 1:1 (*w*/*v*) for washing, centrifuged for 3 min (3000× *g*, 25 °C). The supernatant was collected and then the above process was repeated once. All resulting supernatants, as known as total soy milk, were combined after centrifugation, and then the protein contents of total soy milk was determined by the Kjeldahl method (N × 6.25). After aliquoting the total soy milk, enzymatic hydrolysis and termination reaction were performed according to the method in Section 2.1 and Section 2.2. Before testing, the enzymatic activities of various enzymes were determined. Immediately afterward, the enzymatic hydrolyzed soy milk was passed through a high-pressure homogenizer at 100 bar for one cycle, and then it was subject to spray drying with Inlet air temperature of 185 °C and outlet air temperature of 65 °C at a peristaltic pump speed of 8 rpm. Finally, enzymatic hydrolyzed soy milk powder of trypsin (T-SPH), neutrase (N-SPH), bromelain (B-SPH), papain (P-SPH), flavourzyme (F-SPH) and alcalase (A-SPH) were obtained. The soy milk powder (SP) without enzymatic hydrolysis was used as a control, and ground and refrigerated for later use (Figure 1B).

#### 2.5.1. Soy Milk Powder Solubility Measurements

The soluble soy milk powder content was determined according to Tang et al. with slight modifications [7]. The dispersion was prepared at the soy milk powder concentration of 5.0% (w/v) at 30 °C and stirred for 30 min. Then the dispersion was centrifuged for 15 min (1000× *g*, 20 °C) and carefully removed the supernatant. Additionally, the residual precipitate was dried to a constant weight in an oven at 60 °C prior to analysis. The solubility of soy milk powder was calculated according to the following formula:

(6) $$\%\;S=\frac{W_{ts}-W_{is}}{W_{ts}}\text{×}100$$

where, *W_ts_* is the mass of the solution before centrifugation (g); *W_is_* is the weight of the insoluble solid (g).

#### 2.5.2. Scanning Electron Microscopy (SEM) Measurements

The morphology of the soy milk powder was examined using a Hitachi-S-3400 N scanning electron microscope (Hitachi High Technologies Corp., Tokyo, Japan) at an accelerating voltage of 5 kV. Before the observation under SEM, the soy milk powder was placed on a sample stage and gold coated by means of a sputtering apparatus for electrical conductivity.

#### 2.5.3. Electronic Tongue Evaluation Measurements

Electronic tongue evaluation was determined based on the method described by Weng et al. [16] with some modifications. The soy milk powder was dissolved in distilled water at 70 °C (10%, *w*/*v*), stirred gently for 1 min, and kept warm for 20 min to extract the taste substances before being analyzed using a Taste Sensing System E-tongue (TS-5000Z, Insent Inc., Atsugi-Shi, Japan). The instrument is equipped with five chemical sensors and 1 Ag/AgCl reference electrode, and its chemical sensors is coated with a specific material to respond to different tastes.

### 2.6. Statistical Analysis

All assays were conducted in triplicate, and the results are expressed as the mean ± standard deviation. Statistical analysis involved one-way ANOVA, and Tukey’s post-hoc test with SPSS Statistics version 26 (SPSS Inc., Chicago, IL, USA). A *p*-value of less than 0.05 was used to determine statistical significance.

## 3. Results and Discussion

### 3.1. DH and SDS-PAGE Analysis

**Figure 2:**
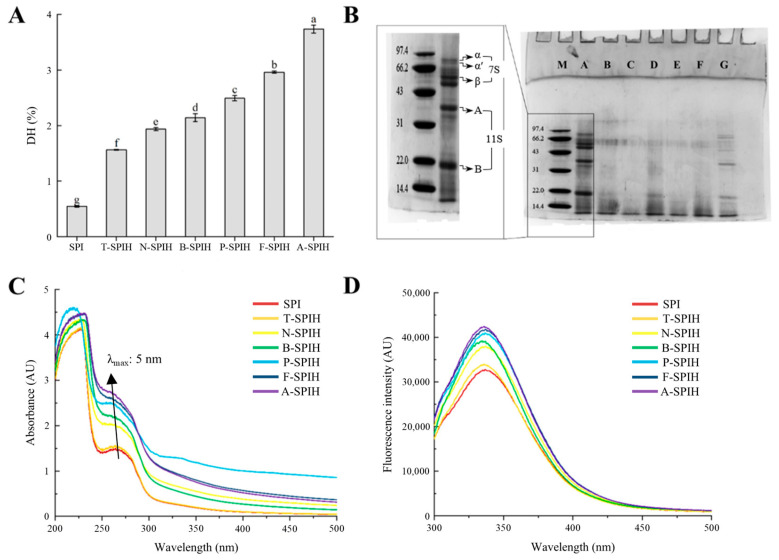
The degree of hydrolysis (**A**), SDS-PAGE analysis (**B**) including: Lane M: low protein molecular weight marker; Lane A: SPI; Lane B: N-SPIH; Lane C: A-SPIH; Lane D: B-SPIH; Lane E: F-SPIH; Lane F: P-SPIH; Lane G: T-SPIH, UV absorbance spectra (**C**), and fluorescence spectra of several protein hydrolysates (**D**). Where, different superscript letters indicate significant differences (same as below).

DH is an important indicator of the degree of protein hydrolysis. As shown in Figure 2A, the SPI exhibited low DH, which are probably due to slight cleavage of its peptide bonds during the preparation process of SPI. Therefore, the DH of SPI did not reflect any enzyme hydrolysis activities [10]. The DH in various hydrolysates exhibited significant differences (*p* < 0.05) following the application of specified treatment conditions. These variations in DH across different enzymes are likely attributable to the enzymes’ specificity, which may be the primary factor contributing to the distinct structural and functional properties observed in the hydrolysates [17]. Among these, the order of enzyme hydrolysis activities is alcalase > flavourzyme > papain > bromelain > neutrase > trypsin. Alcalase exhibits the highest DH, which is due to the extensive hydrolysis of alcalase [18]. Similar results were found in the study of Zhu et al. [19]. However, the bitter taste due to extensive enzymatic hydrolysis of the protein may affect its application in food [20]. Similar to the results in Figure 2A, it was observed that the protein band intensity of the 7S protein (*α*, *α*′ and *β* subunits) and 11S protein (acidic and basic subunits) in SPI decreased and the low molecular weight distribution increased in Figure 2B, confirming that hydrolysis occurrence [21].

### 3.2. UV Absorption and Fluorescence Spectrum Analysis

As shown in Figure 2C, compared with SPI, enzymatic hydrolysis resulted in a significant increase in UV absorption values and a blue shift of the absorption peak. The unfolding of the structure through SPI as a result of the cleavage of peptide bonds of protein during hydrolysis may lead to the exposure of aromatic chromophores and hydrophobic groups, resulting in increased absorbance and caused a blue shift [22]. Significant variations in the UV absorption spectra of protein hydrolysates might be attributed to the distinct specificity of various enzymes. Figure 2D records the fluorescence spectrum of the protein hydrolysate, in which all protein hydrolysate showed higher fluorescence intensity than SPI. Similar to the UV absorption result, alcalase exhibited greater fluorescence intensity. The observed enhancement in fluorescence intensity induced by alcalase further suggests an increased exposure of fluorescent chromophores, possible which due to the fact that the highest degree of hydrolysis was reached by alcalase, as shown in Figure 2A. Much research indicates that the partial hydrolysis of proteins results in their unfolding, thereby enhancing the likelihood of fluorescent chromophore exposure and subsequently increasing protein fluorescence intensity [23,24,25]. Similarly, Zang et al. [26] observed that the intrinsic fluorescence of rice bran protein is influenced by limited trypsin-mediated hydrolysis, which causes protein structure unfolding and reveals fluorescent chromophores previously buried within the protein.

### 3.3. Free Sulfhydryl Group Content and Surface Hydrophobicity Analysis

Disulfide bonds are covalent bonds formed between sulfhydryl groups, and changes in the content of free sulfhydryl groups are closely related to the function of the protein. As demonstrated in Table 1, the free sulfhydryl content of all protein hydrolysates was significantly elevated compared to that of SPI (*p* < 0.05), with a trend that closely paralleled the DH. Notably, hydrolysates treated with alcalase exhibited the highest levels of free sulfhydryl content. This observed increase can be attributed to two primary factors: firstly, the unfolding of the protein structure, which facilitates the exposure of previously buried sulfhydryl groups on the protein surface. Secondary, it may be due to the potential cleavage of disulfide bonds [27]. Protein functional properties may be improved by increasing free sulfhydryl content [28].

Surface hydrophobicity (H_0_) is one of the important factors that affects the structure and functional properties of proteins. It represents the interaction between protein molecules to a certain extent, as shown in Table 1. H_0_ gradually increases as the current increases through the DH. In contrast, there were sequential significant reductions in enzymatic treatment compared to SPI (*p* < 0.05). The potential reasons for this phenomenon can be attributed to two primary aspects. The initial observation is that mild hydrolysis induces a slight unfolding of the SPI structure. It is hypothesized that this unfolding may initially expose a greater number of hydrophobic sites due to hydrolysis, leading to significant hydrophobic interactions that subsequently mask these hydrophobic patches, thereby resulting in a lower H_0_ [29]. This tendency was also observed in previous studies among trypsin treated lentil protein hydrolysates [30] and alcalase treated porcine plasma protein hydrolysate [31]. The second observation pertains to steric hindrance. As the DH increases, hydrophobic groups that were previously concealed within the protein structure become exposed, leading to increased steric hindrance among molecules. The enzymatic hydrolysis-induced exposure of additional hydrophobic groups may account for the initial rise in surface hydrophobicity. This increased steric hindrance diminishes the extent of reaggregation, thereby causing an increase in H_0_ [32]. These findings are consistent with those reported by Shen et al. [21]. Other study also found that the cleavage sites of different enzymes also affect the H_0_ of protein hydrolysates. When DH is the same, flavourzyme has the smallest impact on H_0_, while alcalase has the greatest impact on H_0_. It is speculated that alcalase preferentially cleaves the peptide bonds of hydrophobic amino acids, resulting in enhanced hydrophobic group interactions [33].

### 3.4. Secondary Structure Analysis

**Figure 3:**
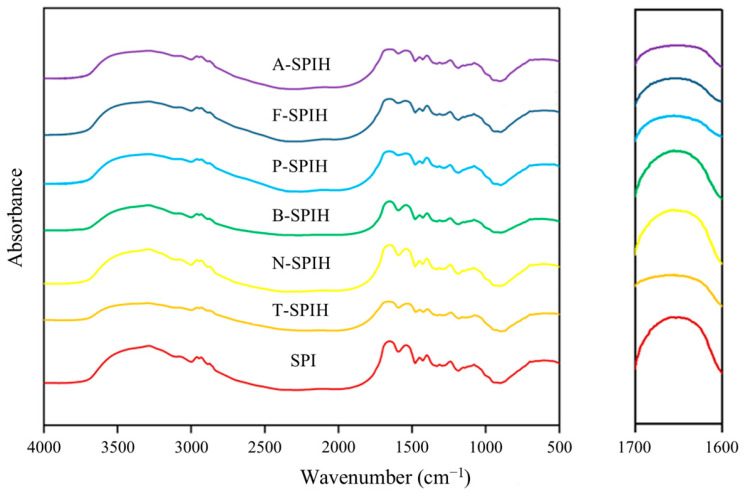
FT-IR spectra of different samples.

Figure 3 presents the FT-IR spectra of various hydrolysates, highlighting the amide I band (1700–1600 cm^−1^) as the most significant feature associated with protein secondary structure. The amide I band corresponds to absorptions related to C=O stretching vibrations. Literature suggests that the C=O groups are instrumental in maintaining the different elements of secondary structure through hydrogen bonding [34]. Observations revealed that the overall amide I absorbance peak became broader and flatter upon treatment in various hydrolysates, indicating alterations in the secondary structures of the proteins as hydrolysis advanced.

A deeper understanding of SPI and their hydrolysates secondary structure was obtained using FT-IR spectra. Table 1 shown the relative secondary structure content of protein hydrolysates induced by different enzymatic treatment. Compared with SPI, enzymatic hydrolysis induced a decrease in *α*-helix and *β*-sheet contents of SPI, whereas the contents of *β*-turn and random coil increased, respectively. The alteration in secondary structure may be attributed to the disruption of hydrogen bonds during the enzymatic hydrolysis process, leading to the exposure of hydrophobic groups on protein side chains [35]. Additionally, the increased presence of random coil indicates a shift towards a more disordered state in protein molecules. The findings indicate that enzymatic hydrolysis may transform the secondary structure of SPI from ordered to disordered, consistent with the results of the free sulfhydryl group content analysis. Similar results were obtained in the study of Zhao et al. [22], who pointed out that hydrolysis led to a significant decrease in ordered structure. However, the secondary structure of the hydrolysate did not maintain the same content after being hydrolyzed by different enzymes. Notably, alcalase exhibited the highest relative content of *β*-turn and random coil, indicating greater flexibility, which is closely associated with protein unfolding and solubility [36].

### 3.5. Solubility Analysis

The primary objective of modifying protein isolates is to enhance solubility, which is a critical prerequisite for improving functionality. As shown in Table 2, enzymatic hydrolysis mediated by enzymes resulted in enhanced solubility of SPI. Generally, the enzymatic hydrolysis of proteins augments the interaction between proteins and water molecules. The increased solubility is attributed to the formation of smaller peptides and the exposure of newly accessible ionizable amino acids and carboxyl groups [37]. Omar et al. [27] also observed a similar phenomenon when using trypsin to hydrolyze sericin. The variation in solubility observed among different protein hydrolysates may be attributed to the diverse sizes of peptide fractions generated during the processes of DH and enzymatic hydrolysis [38]. There are several studies that the solubility of a protein is mainly determined by the surface hydrophobicity [39]. As previously observed by surface hydrophobicity, at the initial stages of hydrolysis, a lower degree of hydrolysis results in exposure of the protein hydrophobic regions, leading to the formation of soluble aggregates through hydrophobic interactions between proteins, thereby enhancing protein-protein interactions [39]. Despite the masking of hydrophobic groups and a reduction in surface hydrophobicity, protein solubility is enhanced due to the formation of these soluble aggregates. As hydrolysis progresses, the degree of hydrolysis increases, leading to the cleavage of soluble aggregates and re-exposure of masked hydrophobic groups on the surface [40]. This results in increased surface hydrophobicity, the generation of more low molecular weight peptides, and further enhancement of protein solubility [6].

### 3.6. Emulsification and Foaming Properties Analysis

As shown in Table 2, different enzymatic hydrolysis showed different EAI increases compared with SPI, among which alcalase showed the greatest improvement. These improvements depended on the enzyme type and its specificity. The enhancement in EAI of protein hydrolysates can be ascribed to the partial unfolding of protein, thereby facilitating protein adsorption at the oil-water interface [41]. In contrast, enzymatic hydrolysis decreased the ESI of SPI. The higher the DH, the more obvious the decrease in ESI. Although these small molecular peptides can diffuse and adsorb on the interface quickly, they are less capable of reducing interfacial tension and stabilizing emulsions because smaller peptides cannot form a stable film around the oil droplets [42]. Similar phenomena were also found in the study of Razavizadeh et al. [43].

Table 2 showed the FC and FS of SPI and its hydrolysates. For FC, beyond alcalase, no differences have been described between other hydrolysates and SPI (*p* > 0.05). Interestingly, this finding agrees with similar observations on the emulsification test, which may be attributed to alcalase contained with a higher DH. Typically, a greater degree of hydrolysis is associated with hydrolysates having a lower molecular weight. As the DH increased, peptide molecules resulting from enzymatic hydrolysis migrated more rapidly to the air–water interface, leading to a more efficient reduction in surface tension, thereby enhancing FC [44]. However, FS results indicated that, in comparison to SPI, the FS of protein hydrolysates initially increased and subsequently decreased with rising DH levels. Lower FS was observed in alcalase and flavourzyme. This may be because the larger DH produced smaller peptide molecules, which hindered the stability of protein molecules on the foam surface, thereby weakening the foaming stability [45]. Hao et al. [46] also found that the FS of SPI was inhibited when the DH reached 5% with alcalase, as the smaller peptide segments in the hydrolysate weakened the film.

### 3.7. Antioxidant Properties Analysis

As shown at Table 2, the DPPH free radical scavenging activity exhibited a significant enhancement with increasing DH (*p* < 0.05), with alcalase demonstrating the most pronounced scavenging capability. The observed enhancement in DPPH radical scavenging activity is likely attributable to the release of small peptide molecules and the exposure of aromatic amino acids and short-chain peptides. [47]. This finding aligns with the results reported by Xu et al. [48], who observed that the DPPH radical scavenging capacity increased with higher DH levels. This increase is potentially due to the release of more bioactive peptides, such as antioxidants, in hydrolysates with elevated DH. The differences in free radical scavenging abilities caused by different enzymes may be attributed to differences in the size, amino acid composition and sequence of the polypeptides produced by the specificity of the enzymes used [42], as well as the content of free sulfhydryl content of protein [49]. On the one hand, the antioxidant activities of the sulfur-containing amino acid, cysteine, is due to their ability to donate protons to free radicals [50]. As shown in Table 1, with the increasing on DH, the free sulfhydryl content increases [51]. This led to significantly enhanced the antioxidant activity for soy protein (*p* < 0.05). On the other hand, the increase in free sulfhydryl content suggested that soy protein became progressively denatured and unfolded as DH increased. The high antioxidant activity of soy protein could be due to the effects of DH aiding the unfolding of soy protein, exposing more sulfhydryl and hydrophobic groups of protein to free radicals, leading to enhanced antioxidant activity [40]. Similarly, a higher ABTS^•+^ free radical scavenging ability was observed in alcalase and flavourzyme. However, no major differences were evident in the response of ABTS^•+^ free radicals (*p* > 0.05), suggesting that their possess similar antioxidant capacity. Studies have shown that peptides with higher DH in hydrolysates may weaken the scavenging activity against ABTS^•+^. This conclusion was supported in the study by Intarasirisawat et al. [52]. However, the different hydrolysates scavenged more ABTS^•+^ free radicals than DPPH free radicals. This variation in activity may be attributed to the differential diffusion abilities of ABTS^•+^ and DPPH free radicals within the reaction medium. ABTS^•+^ free radicals, being soluble in both alcoholic and aqueous solutions, readily interact with peptides in aqueous environments. In contrast, DPPH free radicals are soluble exclusively in alcoholic solutions, thereby impeding the scavenging reaction between DPPH free radicals and peptides [53]. It is important to note that a high capacity to scavenge ABTS^•+^ radicals does not necessarily correlate with a high capacity to quench DPPH radicals [54]. Overall, these results indicate that enzymatic hydrolysis produces hydrolysates with higher antioxidant properties.

### 3.8. Solubility Evaluation of Soy Milk Powder Analysis

**Figure 4:**
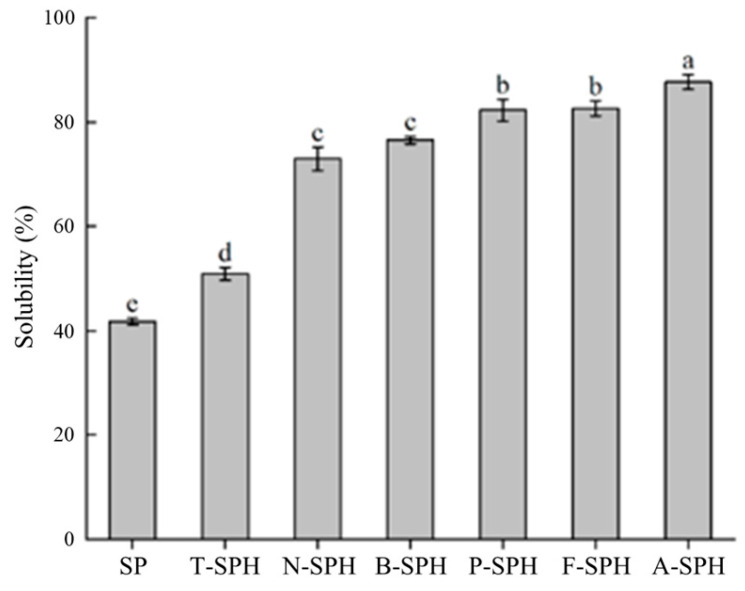
Solubility of hydrolysis of soy milk powder by different enzymes. Where, different superscript letters indicate significant differences (same as below).

Solubility is an important characteristic of soy milk powder. As shown in Figure 4, the solubility of enzymatically hydrolyzed soymilk powder increased significantly compared with SP (*p* < 0.05), with alcalase showing the highest solubility. The improved soy milk powder solubility with enzymatic treatment was attributed to lower protein molecular weight. On the other hand, some studies have indicated that enzyme modification leads to the exposure of additional ionizable amino and carboxyl groups within protein molecules, thereby enhancing protein hydration. In addition, alcalase containing a higher DH may expose more charge and polar amino acid groups of protein, thus further facilitating the solubility of soy milk powder. According to the findings of Zhao et al. [55], an increase in the DH significantly enhances the solubility of egg yolk powder. This improvement can be attributed to the degradation of proteins into peptides with lower molecular weights and increased hydrophilicity, which enhances their interaction with water molecules [56].

### 3.9. SEM Analysis

**Figure 5:**
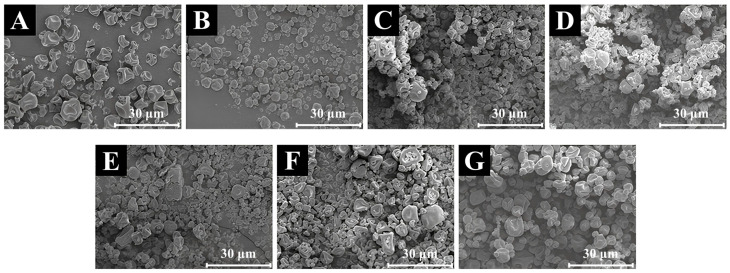
The SEM of hydrolysis of soy milk powder by different enzymes, including: SP (**A**), A-SPH (**B**), F-SPH (**C**), P-SPH (**D**), B-SPH (**E**), N-SPH (**F**), and T-SPH (**G**).

As shown in Figure 5, SEM revealed that the soy milk powder comprised approximately spherical particles with relatively smooth surfaces and compact structures. Some particles displayed noticeable cavities on their surfaces, which may be attributed to the collapse or wrinkling of the particles during the spray drying process [57]. It was observed that as the DH increased, the average size of the hydrolysates decreased. The hydrolysate particles exhibited increased dispersion with reduced aggregation. The introduction of enzymes led to the observation of additional small cavities within the particles, possibly due to the initiation of protein hydrolysis at the particle surface. It is hypothesized that these alterations may enhance the interactions between protein molecules and water, thereby leading to increased solubility. Nevertheless, at higher DH levels (such as flavourzyme and alcalase), the average size was relatively reduced, and particle aggregation was observed. This phenomenon may be attributed to the enzymatic treatment, which exposes a greater number of non-polar groups, thereby facilitating hydrophobic interactions among protein molecules and leading to particle agglomeration [7].

### 3.10. Evaluation of Soy Milk Powder Electronic Tongue

**Figure 6:**
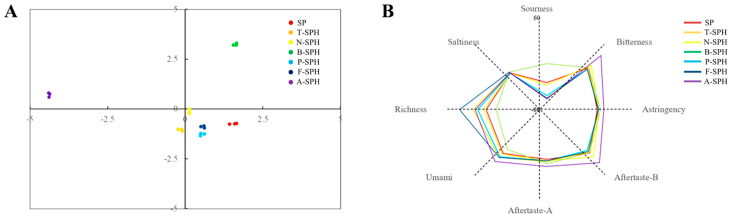
Score chart of flavor principal components (**A**) and radar map (**B**) of soy milk powder.

Figure 6A presents a principal component analysis (PCA) diagram illustrating taste variations as assessed by an electronic tongue. The cumulative variance explained by the first two principal components (PC1 and PC2) is 82.73%, exceeding the threshold of 70%. This suggests that PC1 and PC2 successfully represent the main features of most samples. The PCA model constructed demonstrates robust stability and predictive capability. The samples are distributed across different quadrants with distinct separation and pronounced inter-group differences, suggesting that the incorporation of various enzymes induces alterations in the taste profile of the soy milk powder solution. The observed variations may be attributed to the distinct specificities of the enzymes, which result in differing placements of hydrophobic amino acid residues and the consequent development of diverse flavors. Figure 6B presents a radar chart illustrating the taste profiles of soy milk powder subjected to hydrolysis by various enzymes. enzyme hydrolysis alters the taste of soy milk powder, with a notable enhancement in richness and umami observed in samples treated with flavourzyme. This suggests that the free amino acids generated through flavourzyme hydrolysis contribute to the improved taste profile of the soy milk powder. Flavourzyme, possessing both endo- and exo-peptidase activities, played a crucial role in the preparation of umami protein peptides. This enzyme effectively degrades exposed hydrophobic groups and hydrolyzes specific flavor precursor substances, thereby promoting the formation of high-quality umami products [58]. The bitterness and bitter aftertaste of soy milk powder hydrolyzed by alcalase are significantly higher than those of other samples. This effect probably arises from alcalase’s preference for hydrolyzing at hydrophobic amino acid residues, resulting in the generation of a large amount of bitter peptides, which in turn diminishes the taste quality of soy milk powder [16]. Furthermore, the sour taste of soy milk powder hydrolyzed by bromelain increased significantly, likely due to bromelain’s optimal pH range of 6.5–6.8, which may contribute to this pronounced sourness [59,60]. In summary, the findings indicate that enzymatic hydrolysis can enhance the flavor profile of hydrolyzates, with flavourzyme and alcalase demonstrating superior efficacy in enriching taste. However, it is essential to carefully evaluate the bitterness associated with the use of alcalase.

## 4. Conclusions

This study focused on the effect of promoting the dissolution of soy protein and soy milk powder under different enzymatic hydrolysis. A similar effect has been observed with the enzymatic treatment of six main commercial enzymes for modifying of SPI structure and improving function properties of SPI and soy milk powder. Among the various enzymes studied, alcalase demonstrated significant hydrolytic activity, characterized by a higher DH. Alcalase facilitated the unfolding of SPI, thereby exposing hydrophobic amino acid residues and free sulfhydryl groups, and promoting an increase in disordered structures. Alcalase was identified as the most effective in enhancing the solubility (rose by 190.13%), EAI (484.74%), FC (24.68%), and antioxidant properties of the resulting hydrolysates (DPPH: 144.64%, ABTS^•+^: 91.99%). Concurrently, alcalase significantly improved the solubility of soy milk powder and contributed to a reduction in the particle size of soy milk powder. However, the ESI (decreased by 64.57%) and FS (20.00%) was adversely affected, and the bitterness resulting from extensive hydrolysis persisted as a significant challenge.

## Figures and Tables

**Table 1:** Surface hydrophobicity, free sulfhydryl content, and relative secondary structure content of several protein hydrolysates.

Samples	Surface Hydrophobicity/H_0_	Free Sulfhydryl Group Content/(μmoL/g)	Relative Content of Secondary Structure/%			
			*α*-Helix	*β*-Sheet	*β*-Turn	Random Coil
SPI	38,916.77 ± 384.57 ^a^	3.46 ± 0.05 ^e^	21.82 ± 0.04 ^a^	40.78 ± 0.20 ^a^	19.08 ± 0.02 ^f^	18.33 ± 0.03 ^f^
T-SPIH	14,519.33 ± 729.55 ^f^	3.78 ± 0.03 ^d^	21.19 ± 0.02 ^b^	40.52 ± 0.20 ^ab^	19.79 ± 0.18 ^e^	18.51 ± 0.01 ^e^
N-SPIH	20,299.16 ± 260.15 ^e^	3.87 ± 0.03 ^d^	18.64 ± 0.03 ^c^	40.22 ± 0.09 ^b^	20.44 ± 0.18 ^d^	20.72 ± 0.03 ^d^
B-SPIH	26,565.35 ± 401.54 ^d^	5.28 ± 0.03 ^c^	17.09 ± 0.01 ^d^	38.12 ± 0.09 ^c^	23.95 ± 0.07 ^c^	20.84 ± 0.07 ^d^
P-SPIH	29,415.68 ± 1097.15 ^c^	5.59 ± 0.04 ^b^	17.20 ± 0.01 ^d^	37.36 ± 0.15 ^d^	24.15 ± 0.09 ^c^	21.28 ± 0.18 ^c^
F-SPIH	30,365.79 ± 1555.24 ^c^	5.68 ± 0.07 ^b^	16.79 ± 0.07 ^e^	36.02 ± 0.14 ^e^	25.28 ± 0.10 ^b^	21.90 ± 0.10 ^b^
A-SPIH	34,403.75 ± 576.85 ^b^	7.37 ± 0.07 ^a^	15.11 ± 0.17 ^f^	34.51 ± 0.21 ^f^	26.66 ± 0.02 ^a^	23.73 ± 0.09 ^a^

Where, different superscript letters indicate significant differences (same as below).

**Table 2:** The solubility, emulsifying and foaming properties, and antioxidant activity of several protein hydrolysates.

Samples	Solubility/%	EAI/(m^2^/g)	ESI/%	FC/%	FS/%	DPPH/%	ABTS/%
SPI	6.89 ± 1.18 ^e^	14.88 ± 1.32 ^d^	37.77 ± 5.02 ^a^	1.58 ± 0.05 ^c^	0.50 ± 0.09 ^cd^	21.08 ± 0.27 ^f^	43.46 ± 0.46 ^e^
T-SPIH	9.45 ± 0.94 ^d^	17.88 ± 0.90 ^d^	20.35 ± 0.61 ^b^	1.63 ± 0.09 ^bc^	0.97 ± 0.03 ^a^	40.63 ± 0.79 ^e^	67.16 ± 1.61 ^d^
N-SPIH	13.39 ± 0.68 ^c^	31.35 ± 5.83 ^c^	15.82 ± 1.27 ^bc^	1.65 ± 0.10 ^bc^	0.72 ± 0.02 ^b^	45.50 ± 0.97 ^d^	67.61 ± 1.10 ^d^
B-SPIH	15.55 ± 0.91 ^bc^	42.43 ± 7.45 ^c^	14.34 ± 0.71 ^c^	1.79 ± 0.11 ^abc^	0.67 ± 0.04 ^b^	47.62 ± 0.25 ^c^	71.43 ± 0.74 ^c^
P-SPIH	17.06 ± 1.20 ^b^	59.71 ± 3.28 ^b^	13.95 ± 0.45 ^c^	1.80 ± 0.17 ^abc^	0.62 ± 0.06 ^bc^	47.77 ± 0.62 ^c^	82.14 ± 1.44 ^b^
F-SPIH	17.09 ± 0.22 ^b^	78.54 ± 5.66 ^a^	13.14 ± 1.26 ^c^	1.87 ± 0.11 ^ab^	0.37 ± 0.04 ^e^	49.81 ± 0.17 ^b^	85.71 ± 0.40 ^a^
A-SPIH	19.99 ± 0.86 ^a^	87.01 ± 4.03 ^a^	13.38 ± 1.16 ^c^	1.97 ± 0.10 ^a^	0.40 ± 0.03 ^de^	51.57 ± 0.17 ^a^	83.44 ± 1.68 ^ab^

Where, different superscript letters indicate significant differences (same as below).

## Data Availability

The original contributions presented in the study are included in the article, further inquiries can be directed to the corresponding authors.
